# The Neurophysiological Responses of Concussive Impacts: A Systematic Review and Meta-Analysis of Transcranial Magnetic Stimulation Studies

**DOI:** 10.3389/fnhum.2020.00306

**Published:** 2020-08-27

**Authors:** Emily Scott, Dawson J. Kidgell, Ashlyn K. Frazer, Alan J. Pearce

**Affiliations:** ^1^College of Science, Health and Engineering, La Trobe University, Melbourne, VIC, Australia; ^2^Department of Physiotherapy, Faculty of Medicine, Nursing and Health Science, School of Primary and Allied Health Care, Monash University, Melbourne, VIC, Australia

**Keywords:** concussion, transcranial magnetic stimulation, evoked potentials, motor, systematic review, meta-analysis

## Abstract

**Aim:** This systematic review and meta-analysis investigated neurophysiological responses using transcranial magnetic stimulation (TMS) following a concussion or sub-concussion.

**Methods:** A systematic searching of relevant databases for peer-reviewed literature quantifying motor evoked potentials from TMS between 1999 and 2019 was performed. A meta-analysis quantified pooled data for measures including motor threshold, motor latency, and motor evoked potential amplitude and for inhibitory measures such as cortical silent period duration, short-interval intracortical inhibition (SICI), and long-interval intracortical inhibition (LICI) ratios.

**Results:** Fifteen articles met the inclusion criteria. The studies were arbitrarily classified into the groups, based on time post-concussion, “acute” (subjects 0–3 months post-injury, *n* = 8) and “post-acute” (3 months−2 years post-concussion, *n* = 7). A TMS quality of study checklist rated studies from moderate to high in methodological quality; however, the risk of bias analysis found that the included studies were categorised as high risk of bias, particularly for a lack of allocation concealment and blinding of participants in the methodologies. A meta-analysis showed no differences in excitability measures, apart from a decreased motor threshold that was observed in the concussed group (SMD −0.28, 95% CI −0.51 to −0.04; *P* = 0.02) for the post-acute time frame. Conversely, all inhibitory measures showed differences between groups. Cortical silent period duration was found to be significantly increased in the acute (SMD 1.19, 95% CI 0.58–1.81; *P* < 0.001) and post-acute (SMD 0.55, 95% CI 0.12–0.98; *P* = 0.01) time frames. The SICI (SMD −1.15, 95% CI −1.95 to −0.34; *P* = 0.005) and LICI (SMD −1.95, 95% CI −3.04 to −0.85; *P* = 0.005) ratios were reduced, inferring increased inhibition, for the post-acute time frame.

**Conclusion:** This systematic review and meta-analysis demonstrates that inhibitory pathways are affected in the acute period post-concussion. However, persistent alterations in cortical excitability remain, with increased intracortical inhibition. While TMS should be considered as a reliable technique to measure the functional integrity of the central nervous system, the high risk of bias and heterogeneity in data suggest that future studies should aim to incorporate standardised methodological techniques, particularly with threshold determination and stimulus intervals for paired-pulse measures.

## Introduction

Concussive brain injury is a global health issue (World Health Organisation, 2006) affecting a broad range of people who experience accidents in workplaces, home environments, and road traffic incidents (Koerte et al., 2015; Lefebvre et al., 2015). However, concussions experienced in sport, while historically under-recognised and underreported, have received increased attention by the public over recent years due to growing media attention (Lefebvre et al., 2015; Coyle et al., 2018). Resultantly, there have been increased investigations studying the underlying neurological mechanisms to inform the development of diagnostic, monitoring, and treatment protocols (Koerte et al., 2015; Lefebvre et al., 2015; Kamins et al., 2017). While much of this culture shift has been catalyzed by the publishing of neuropathological and long-term neurological impairment studies in retired professional athletes (Pearce et al., 2014, 2018, 2020b; Koerte et al., 2015; Buckland et al., 2019; Mez et al., 2019), there is a push to elicit explanations of the shorter-term impacts and underlying pathophysiology (Lefebvre et al., 2015) that can determine long-term sequelae.

Concussion has been defined as a functional traumatic brain injury (McCrory et al., 2017). The absence of findings on medical imaging (Dimou and Lagopoulos, 2014; McCrory et al., 2017; Coyle et al., 2018) are interpreted as a lack of structural damage. Despite there being no findings on brain imaging scans, the subjects experience a raft of symptoms post-concussion, such as cognitive fatigue, light and noise sensitivity, and reduced cognitive processing capacity and executive functioning (Johansson et al., 2009; Johansson and Rönnbäck, 2012, 2014; Koerte et al., 2015; Coyle et al., 2018). These symptoms have rather been described as reflecting functional changes on a cellular metabolic level (Giza and Hovda, 2001, 2014; McCrory et al., 2017; Coyle et al., 2018). For example, Giza and Hovda (2001, 2014) describe a pathophysiological cascade whereby variations in metabolism and cerebral blood flow place stress in neuronal functioning in the acute phase (7–10 days) following injury (Giza and Hovda, 2001, 2014). This neurobiological response to concussion is not only highly dependent on the time post-interval but may also affect brain function in the post-acute phase in the weeks and months post-concussion. Of growing concern, however, is the emerging evidence of neurophysiological changes persisting beyond the resolution of symptoms (Kamins et al., 2017). As self-reported symptom heavily informs clinical judgment for medical clearance to return to normal life activities, including sports, school, and work participation, people may be returning before the brain has appropriately recovered, placing them at risk of further injury (Koerte et al., 2015; Kamins et al., 2017). However, the implications of post-concussive morbidity are outside the scope of this review.

Neurophysiological measures provide the opportunity to quantify the functional disturbances in concussion as suggested by McCrory et al. (2017), potentially informing the physician of subtle and prolonged changes in brain physiology in light of symptom resolution. Techniques such as electroencephalography (EEG) and transcranial magnetic stimulation (TMS) have shown alterations in evoked potentials despite the individuals being asymptomatic. A recent systematic review in 16 studies presented some abnormalities in resting state EEG activity following concussion but noted variability in affected cortical rhythms, reflecting methodological and analytical differences between study designs (Conley et al., 2019). EEG studies have also been utilised in studies of sports with high volume of “sub-concussive” trauma, defined as where the brain experiences impacts such as soccer heading, bumps or tackles in football, or punches in boxing, but without the overt signs or symptoms of concussion (Erlanger, 2015). A systematic review by Tarnutzer et al. (2017) reported two studies investigating sub-concussion via EEG; one study in the review by Tarnutzer et al. (2017) reported abnormal EEG activity in players who self-reported as “non-headers,” compared to players who considered themselves “headers” of the ball (Tysvaer and Storli, 1989), whereas another study showed no EEG differences in headers and non-headers (Tysvaer et al., 1989).

Developed in 1985 (Barker et al., 1985a,b), TMS is a well-established technique, providing reliable measures of corticomotor excitation and inhibition of the primary motor cortex (M1), the spinal nerve roots, and the peripheral motor pathway across a range of healthy, experimental, and diseased conditions (Hallett, 2000; Kobayashi and Pascual-Leone, 2003; Rossini and Rossi, 2007). For example, TMS has been used to measure corticomotor excitability following exercise and strength training in healthy populations (Kidgell and Pearce, 2010; Kidgell et al., 2017), while TMS has also been utilised to detect subject changes across a range of neurological conditions (Kobayashi and Pascual-Leone, 2003) as well as psychiatric disorders (Bunse et al., 2014) and also in intriguing conditions such as vascular cognitive impairments (Lanza et al., 2017) and celiac disease (Pennisi et al., 2017).

TMS employs time-varying magnetic fields that induce electrical currents in conductive neural tissue. When applied over the M1, the response is recorded and measured as a motor evoked potential (MEP) in the electromyogram (EMG) of the target muscle (Hallett, 2000; Pearce and Morris, 2011). The MEP measures from TMS–EMG have been previously well-described by Hallett (2000) and Kobayashi and Pascual-Leone (2003). Single-pulse TMS measures include resting (rMT) and active motor threshold (aMT), measures of excitation, and quantifying the magnitude of stimulation required to excite a muscle fibre (Chen, 2000; Lefebvre et al., 2015). Latency represents the time from stimulus (e.g., TMS impulse) to onset of muscle activation (MEP) and is also an excitatory measure (Lefebvre et al., 2015). MEP amplitude is comprised of descending volleys generated by direct (D-waves) and indirect (I-waves) synaptic activation of corticospinal neurons, reflecting excitability in both primary motor cortex and the spinal cord, and is typically considered a measure of corticospinal excitability (Chen, 2000). The MEP is usually measured from the peak-to-peak of the waveform and expressed either as a raw amplitude in mV, ratio of peripheral M-wave amplitude as %, MEP_MAX_, or arbitrary units from a stimulus–response curve (Pearce et al., 2013). Cortical silent period (cSP), a measure of intracortical inhibition, is quantified as the duration from the onset of MEP waveform to the return of uninterrupted sEMG activity, mediated by the neurotransmitter γ-aminobutyric acid type B (GABA_B_) (Wilson et al., 1993; Hallett, 2000; Kobayashi and Pascual-Leone, 2003). Paired-pulse TMS allows for an assessment of the physiology of the intrinsic intra-cortical connections (Chen, 2000; Hallett, 2000; Lefebvre et al., 2015) and includes SICI and LICI, calculated as the ratio of the test stimulus and the conditioning stimulus (Kujirai et al., 1993; Chen, 2000; Di Lazzaro et al., 2004). As a result of increasing interest in using TMS, the most recent consensus statement for concussion in sport has included TMS as an appropriate technique to measure the neurophysiology of concussion (McCrory et al., 2017).

Previous narrative and qualitative systematic reviews have suggested that, following concussion, the inhibitory motor system is disrupted (Lefebvre et al., 2015; Major et al., 2015). To date there is no meta-analytical evidence that has quantified the effect of concussion via TMS responses. Consequently, the strength of evidence regarding the acute and the post-acute effects of concussion on corticomotor excitatory and inhibitory pathways has not yet been undertaken.

In order to make progress toward better diagnostics and management of concussion, an increased understanding of the underlying pathophysiology is crucial (Koerte et al., 2015; Lefebvre et al., 2015; Coyle et al., 2018). Therefore, the aim of this systematic review and meta-analysis was to systematically determine the effect of concussive and sub-concussive injury on specific TMS responses in order to identify trends in neurophysiological parameters. This review focused on data collected in the acute (0–3 months post-injury) and post-acute (3 months−2 years post-injury) phases post-head trauma. We hypothesised that concussed individuals would display altered corticomotor physiology, specifically via increased cortical inhibition and decreased cortical excitability, when compared with age-matched healthy controls.

## Methods

The research question was developed using the population, intervention, comparison, and outcome model, in agreement with the Preferred Reporting Items for Systematic Reviews and Meta-analysis (PRISMA) statement (Moher et al., 2015). Our research question specifically asked what are the corticomotor excitation and inhibition changes in athletes following concussion (P, contact sport athletes; I, concussion or mTBI, without transcranial stimulation intervention, see “Criteria for Inclusion and Exclusion of Articles”; C, non-concussed athletes; O, corticomotor excitation and inhibition via TMS).

### Search Strategy

**Figure 1:**
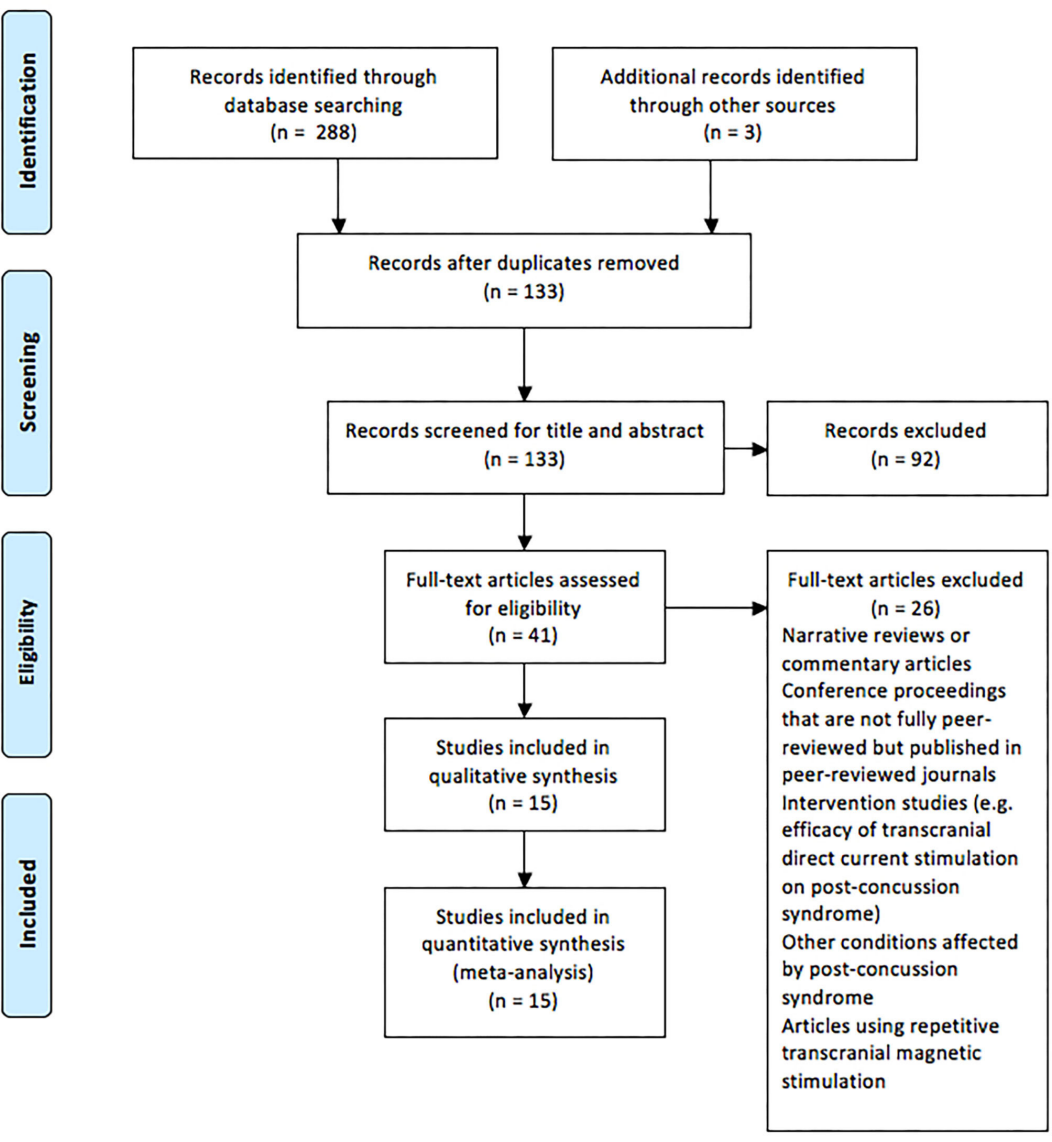
Preferred Reporting Items for Systematic Reviews and Meta-analysis flow chart of the studies included in the review.

During September 2019, the following databases were searched: Web of Science, Current Contents, Medline, PubMed, Scopus, and SPORTDiscus. Boolean operators (AND, OR) were used in various combinations for the following medical subject headings (MeSH) and non-MeSH terms: “brain concussion,” “mild traumatic brain injury,” “cerebral concussion,” “commotio cerebri,” “concussion, mild,” “concussion,” “transcranial magnetic stimulation,” “transcranial magnetic stimulation, single pulse,” “transcranial magnetic stimulation, paired pulse,” and “evoked potentials, motor.” Duplicate articles were then removed, and the titles and the abstracts of search results were screened following the application of criteria according to the PRISMA guidelines (Moher et al., 2009), as outlined in Figure 1. Full-text PDFs of the articles were obtained and exported with their citations into Endnote X9 (Thomas Reuters, New York, USA), with no further modifications of these references.

### Criteria for Inclusion and Exclusion of Articles

Each database search was limited to peer-reviewed, full-text publications printed in English between 1 January 1999 and 17 December 2019. Further inclusion criteria were (i) human studies, (ii) subjects over 19 years in age, (iii) randomised control trials, quasi-experimental studies, observational and comparative studies with controls, case series, and systematic reviews (with and without meta-analysis), and (iv) studies with post-concussion TMS–EMG measures within 2 years of the individual's concussion.

The exclusion criteria applied to each search included (i) non-peer or limited review conference proceedings, (ii) conference abstracts, (iii) books, (iv) theses (PhD, masters, honors), and (v) studies where single- or paired-pulse TMS was not the main technique. For example, data from related techniques such as transracial direct current stimulation (Wilke et al., 2017), repetitive TMS paradigms including theta-burst protocols measuring the intervention via TMS–EMG/TMS–EEG (Moussavi et al., 2019; Opie et al., 2019), or paired associative stimulation (De Beaumont et al., 2012a) were used as interventions to explore other aspects of maladaptive plasticity, such that the influence of brain-derived neurotrophic factor in implicit learning (De Beaumont et al., 2012b) was excluded as this was outside of the scope of this review.

### Allocation of Studies, TMS Quality Analysis, and Risk of Bias

In order to quantify the acute and the post-acute time-phase neurophysiological effects of concussion and sub-concussion in the primary motor cortex (M1), studies utilising TMS were grouped into the following time-specific arbitrary categories: “acute” and “post-acute.” “Acute” included studies from immediately after injury to 3 months post-injury. “Post-acute” referred to studies >3 months– <2 years post-injury. The author's judgment was used to classify studies that had time points from at least two categories. Emerging evidence suggests that the neurophysiological effects of concussion and sub-concussion may differ with time post-injury, which was the rationale for dividing studies in this manner (De Beaumont et al., 2007; Miller et al., 2014; Pearce et al., 2014, 2015; Di Virgilio et al., 2016).

A checklist was used to assess the TMS methodological quality of studies (Chipchase et al., 2012). The items contained in the checklist addressed specific items in studies across broad areas:

- Participant factors (age, gender, handedness)
- Clinical factors (reporting, if applicable, of medical conditions or neurological/psychiatric conditions, or medications that the participants were currently prescribed with)
- TMS protocol factors (such as position of electromyography electrodes, contraction intensity during stimulation, TMS coil type, location over the scalp, orientation of coil, stimulation intensity, time between MEP pulse, and pulse type)
- Single and paired-pulse MEP measures [such as the normalisation of MEP amplitude between participants and, for the paired-pulse, the intensity of the conditioning and test pulses, and inter-stimulus intervals for short-interval intracortical inhibition (SICI) and long-interval intracortical inhibition (LICI)].

Each question in the checklist was assessed for “reported” and “controlled” with half a mark given for each check (except for “gender” and “level of relaxation of muscles other than those being tested” which were not applicable for “controlled” or “reported,” respectively). The maximum score for single pulse studies was 26.

The methodological quality of all included studies was assessed by two authors independently (DJK and AKF) using the Cochrane Collaboration of risk-of-bias tool. Each study was scored for six potential sources of bias: sequence generation, allocation concealment, blinding of participants and personnel, blinding of the outcome assessors, incomplete outcome data, and selective reporting. A rating of “low” or “high” was assigned if the criteria for a low or high risk of bias were met, respectively. The risk of bias was judged “unclear” for a domain if inadequate details were reported or if what happened in the study was known but the risk of bias was still uncertain. Disagreement between authors regarding the risk of bias was resolved by consensus.

### Data Extraction and Analyses

For all the included articles, data extraction involved the retrieval of study characteristics (author, year, sample size, and study design), participant demographics (age, sex), time post-concussion (acute, post-acute), and total number of concussions accumulated. TMS variables included motor threshold, latency, MEP amplitude, cSP duration, SICI, and LICI. In all but one study (Davidson and Tremblay, 2016), which reported data in both hemispheres, TMS measures were analyzed from the dominant limb muscle.

Where mean ± SD or SE values were not provided for post-intervention parameters, raw data (means and SD) were derived or calculated from SE, 95% confidence intervals (CI), *P*-values, *t*-values, or *F*-values. Furthermore, when only graphs were available in text, data were extracted from the graphs with the Plot Digitizer software (V4.2, San Francisco, CA, USA), a freeware program for extracting data presented in papers as linear, logarithmic axis scales, and scatter plots).

### Statistical Analysis

The post-concussion data from the concussion injury and control groups for each study were used for the following variables: MEP excitability, cSP, SICI, and LICI. As systematic influences and random error were predicted to be present between study level effect sizes, a random effects meta-analysis was performed to compare the overall pooled SMDs for the main outcome measures (Borenstein et al., 2010). SMDs with 95% confidence intervals were used to measure the intervention effect as the included studies presented outcome measures in a variety of ways. SMD values of 0.20 ≤ 0.49 indicated small, 0.50 ≤ 0.79 indicated medium, and ≥0.80 indicated large effects (Cohen, 2013). For outcome measures for which studies were found to be highly homogeneous and employed the same units of measurement as well as consistent methodological procedures for the electrophysiological recordings, the mean difference (MD) of the changes along with its SD was used to obtain an absolute estimate of effect. To examine the extent of variation among study effects (between-study variance), *t*^2^ and chi-square test, along with the *I*^2^ analysis, were used. The *I*^2^ statistic was used to indicate the percentage variance between studies, with cutoff points corresponding to low (25%), moderate (50%), and high (75%) heterogeneity. In case of heterogeneity exceeding this threshold, a leave-one-out sensitivity analysis was performed to check whether our findings were driven by a single study. Funnel plots assessed publication bias and were inspected visually. All statistical analyses were performed in RevMan 5.3 (Deeks et al., 2011) using an alpha level of *P* < 0.05.

## Results

Figure 1 displays the PRISMA flow chart showing the process of study identification, screening, and evaluation of the eligibility of the included studies. The initial search yielded 288 titles based upon titles and abstracts. Additional searching brought up further records (*n* = 3). Following the removal of duplicates, the abstracts and the titles of the remaining 133 records were screened, with 92 publications removed as they did not meet the eligibility criteria. Full-text papers (*n* = 41) were assessed for eligibility, with a further 26 of these being removed. Therefore, 15 articles were included for analysis (Table 1).

**Table 1:** Overview of included studies.

**References**	**Groups**	**Mean age (years)**	***M***	***F***	**Mean time since injury data collected**	**Mean number of concussions**	**Additional assessments reported**	**TMS quality study score**
Christyakov et al. (2001)	Concussion Control	31.9 33.2	9 15	5	2 weeks	N/A	GCS 13–15	23
Davidson and Tremblay (2016)	Concussion Control	24.3 ± 3.1 24.4 ± 4.8	12 12	4 4	17 months	2	Neuropsychological Motor performance test	21
De Beaumont et al. (2011)	Concussion Control	22.3 ± 3.4 22.3 ± 3.4	21 15	0	19.03 ± 13.77 months	2.65 ± 1.45	Postural stability Motor execution speed	17
De Beaumont et al. (2012b)	Concussion Control	23.4 ± 3.1 23.4 ± 3.1	13 19	0	13.74 ± 6.26 months	2.87 ± 1.41	GCS 13–15/Motor learning task	18
Di Virgilio et al. (2019)	Sub-concussion Control	22 ± 1.7 22 ± 3	NS	NS	1 h post	0 (sub concussive intervention)	Postural control Neuropsychological Electromyography	18
Edwards and Christie (2017)	Concussion Control	20.8 ± 2.3 20.9 ± 0.9	5 7	4 7	2, 4, and 8 weeks	NS	Post-concussion symptoms/Neuropsychological	15
Livingston et al. (2010)	Concussion Control	20.4 ± 1.3 20.4 ± 1.3	6 6	3 3	1 day	NS	Graded concussion severity	17
Miller et al. (2014)	mTBI Control	20.8 ± 1.2 21.1 ± 1.3	8 8	7 7	2.6 ± 0.2 days and 8 weeks	<2	Brief medical history	22
Pearce et al. (2015)	Concussion Control	25.0 ± 2.6 25.2 ± 4.4	8 15	0 0	5 days	N/A	Neuropsychological Fine motor dexterity Visuomotor reaction time	24
Pearce et al. (2019)	PPCS Asymptomatic Control	36.2 ± 14 33.8 ± 6.6 37.7 ± 8	15 16 16	5 4 4	15.6 ± 7.6 months 12.5 ± 6.6 months	4.0 ± 3.0 4.8 ± 2.6	Self-report fatigue scale Somatosensory vibration	22
Pearce et al. (2020a)	PPCS Asymptomatic	39.7 ± 13.5 36.3 ± 9.5	33 41	5 4	14.1 ± 7.1 months 12.5 ± 6.9 months	4.6 ± 3.5 4.7 ± 2.6	Self-report fatigue scale Somatosensory vibration	19
Powers et al. (2014)	Concussion Control	20.2 ± 1.2 20.3 ± 1.5	8 8	0 0	34 days	N/A	Voluntary activation Sense of force	22
Stokes et al. (2020)	Concussion (acute) Concussion (chronic) Control	All participants 18–22 years	12 21 29	0 0 0	<2 weeks >1 year	N/A N/A	IQ Concussion history survey Concussion symptom survey	20
Tremblay et al. (2011)	Concussion Control	22.4 ± 1.7 23.2 ± 5.9	12 14	0 0	23.2 ± 5.9 months	3.2 ± 0.9	Electroencephalography	23
Yasen et al. (2017)	Mtbi Control	21.2 ± 4.4 21.4 ± 4.6	10 10	10 10	72 h and 8 weeks	1	Post-concussion symptoms Cognitive testing Gait analysis	23

*Data displayed as mean (± SD) where reported*.

*NS, Not stated; N/A, Not applicable; GCS, Glasgow Coma Scale; IQ, Intelligence Quotient*.

### Quality Assessment

**Figure 2:**
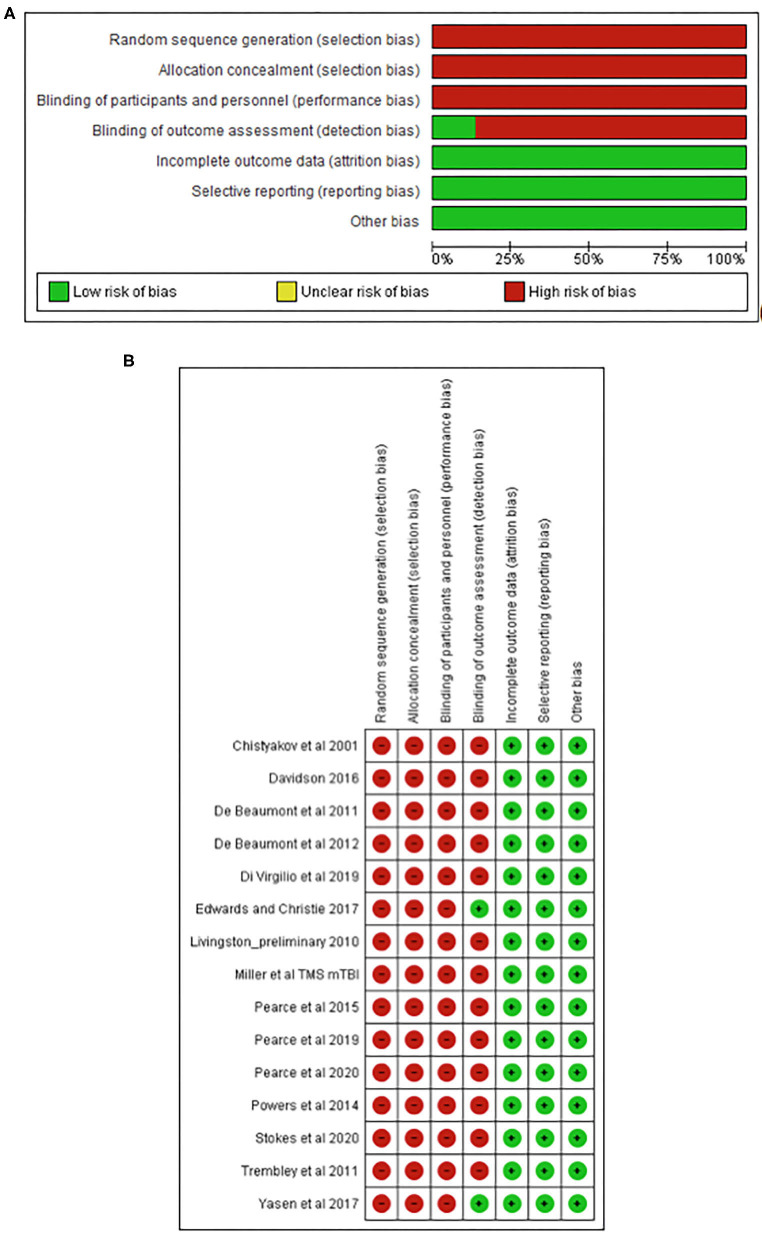
Risk of bias graph **(A)** and study summary **(B)** review authors' judgments about each risk of bias item presented as percentages across all included studies.

The TMS study checklist (Table 1) ranged between 15 and 25, making studies of moderate to high methodological quality. The included studies were categorised as “high risk of bias” for random sequence generation, allocation concealment, and blinding of participants and personnel; however, “low risk of bias” was found for attrition and reporting. The detailed results from the risk of bias assessment using the Cochrane risk of bias tool (Deeks et al., 2011) are presented in Figures 2A,B.

### Motor Threshold and MEP Latency

**Figure 3:**
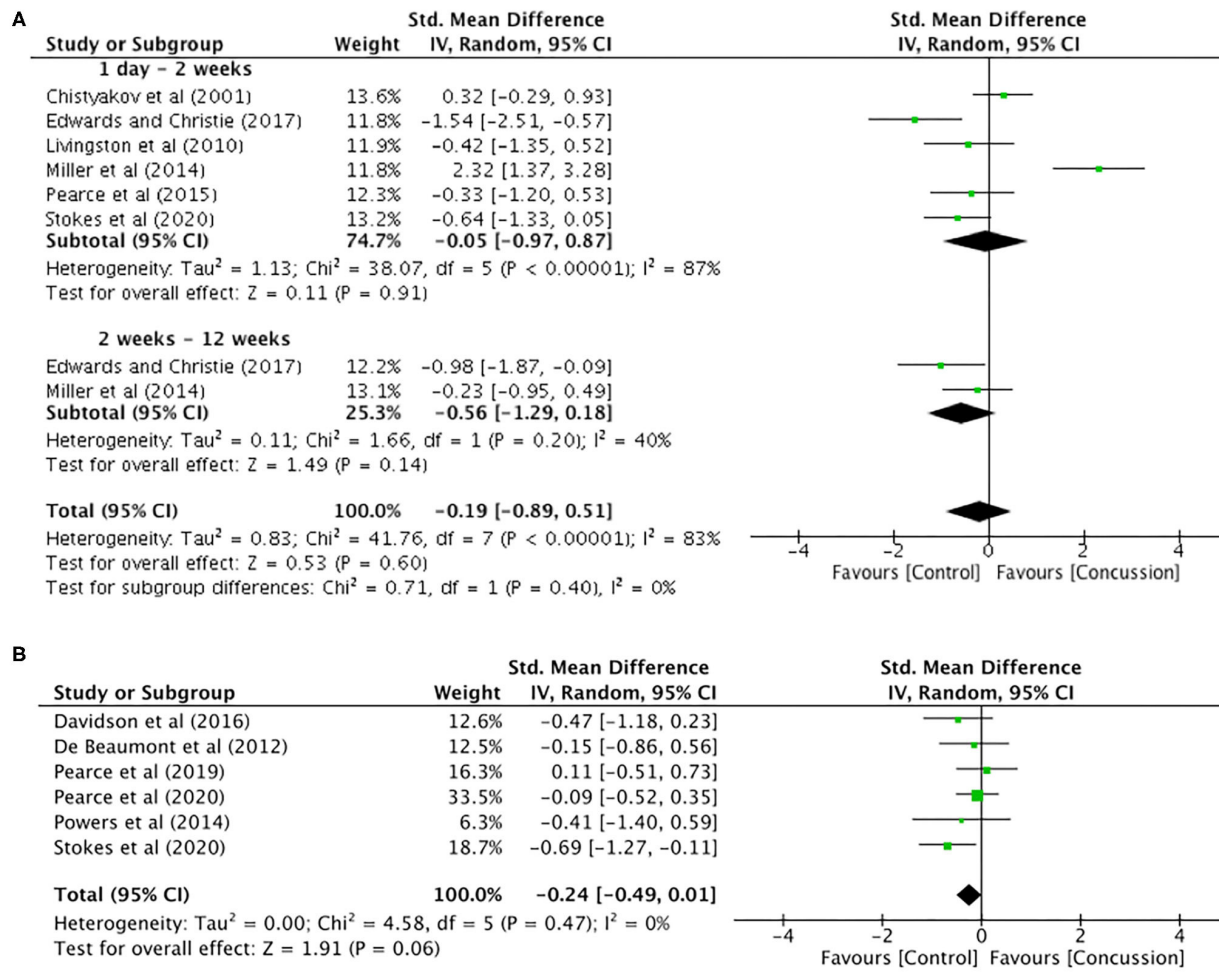
Motor threshold data, 1 day−12 weeks post-concussion **(A)** and 12 weeks−2 years post-concussion **(B)**.

Motor threshold data were extracted from six studies for data up to 12 weeks (concussed, *n* = 99; controls, *n* = 131; Figure 3A). The pooled data indicated that, following a concussion, no change in motor threshold was observed (SMD −0.19, 95% CI −0.89–0.51; *P* = 0.02; Figure 3B) and the heterogeneity across studies was high (τ^2^ = 0.83; χ^2^ = 41.76; *df* = 7; *P* < 0.001; *I*^2^ = 83%). The pooled data for motor threshold in studies between 12 weeks and 2 years (Figure 3B) showed no significant differences between concussed (*n* = 116) and controls (*n* = 137) participants (SMD −0.24, 95% CI −0.49–0.01; *P* = 0.06; Figure 3B), and the heterogeneity across studies was low (τ^2^ = 0.00; χ^2^ = 4.58; *df* = 5; *P* = 0.47; *I*^2^ = 0%).

**Figure 4:**
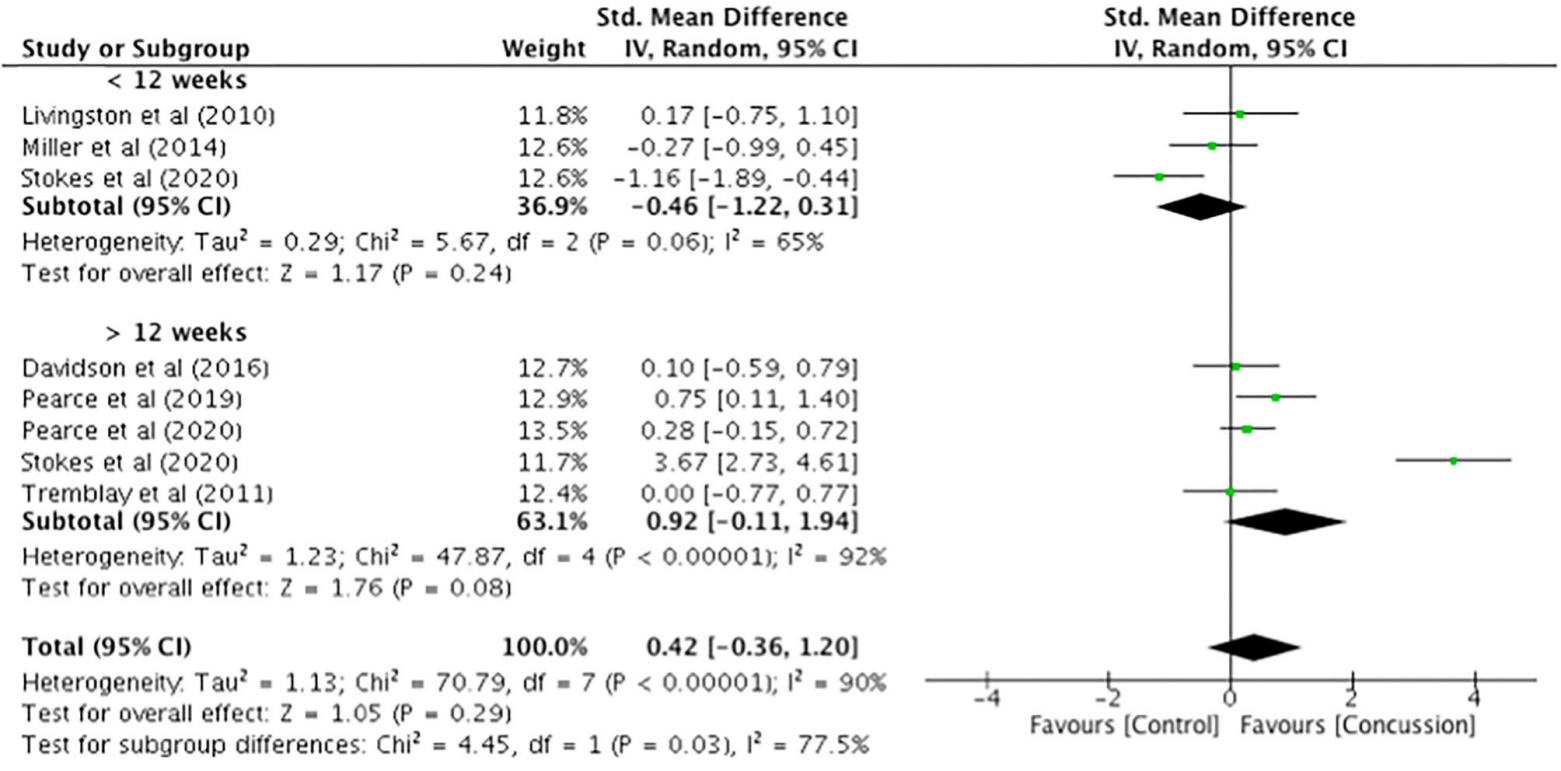
Latency data, <12 weeks and 12 weeks−2 years post-concussion.

MEP latency (Figure 4) was extracted from seven studies. Comparisons between groups (concussed, *n* = 143; controls, *n* = 177) showed no differences in latency (SMD 0.42, 95% CI −0.36–1.20; *P* = 0.29), and the heterogeneity across studies was high (τ^2^ = 1.13; χ^2^ = 70.79; *df* = 8; *P* < 0.0001; *I*^2^ = 90%).

### MEP Amplitude

**Figure 5:**
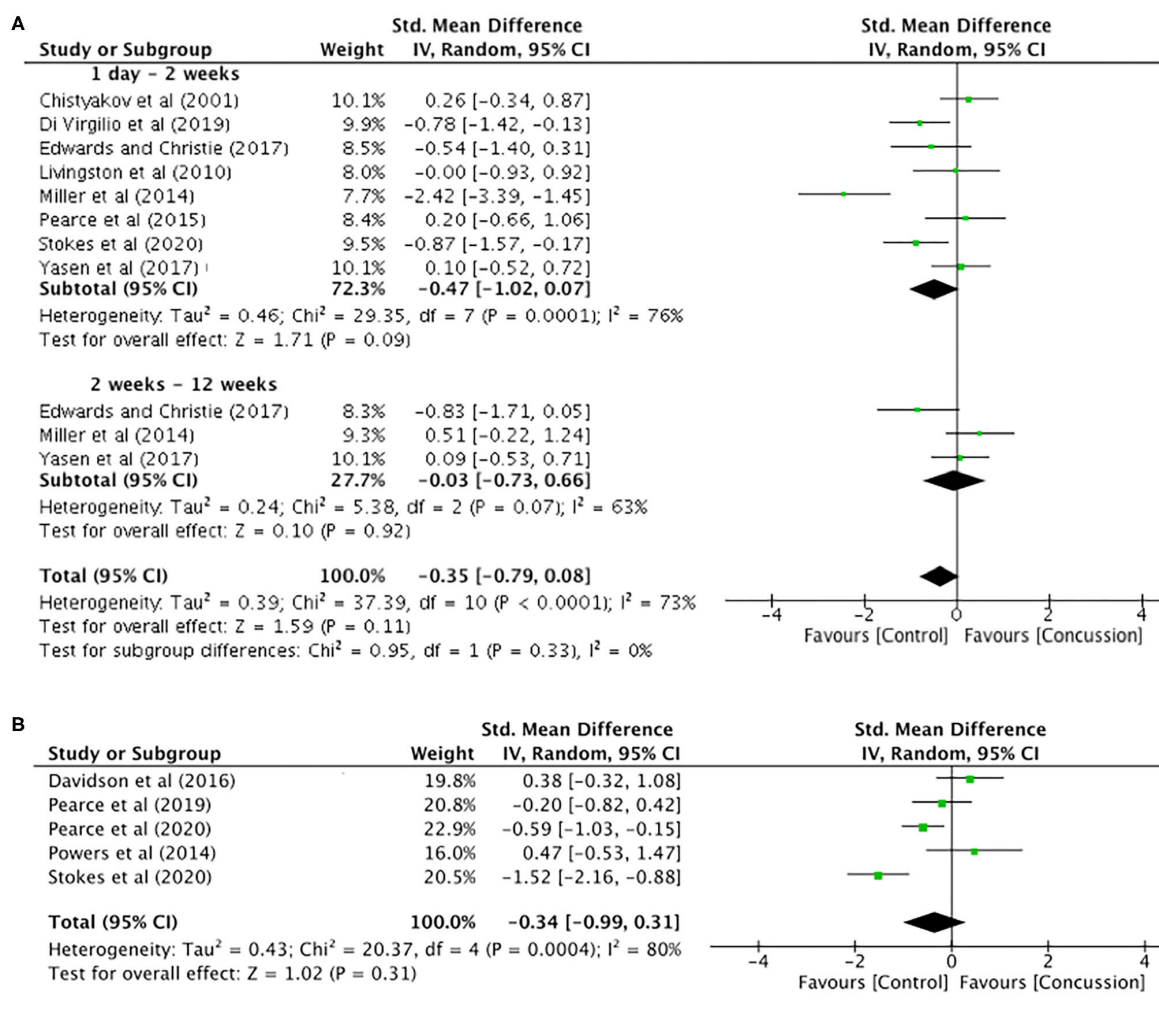
Motor evoked potential amplitude data, 1 day−12 weeks post-concussion **(A)** and 12 weeks−2 years post-concussion **(B)**.

MEP amplitude data up to 12 weeks post-concussion for eight studies are presented in Figure 5A. The data extracted up to 2 weeks post-concussion did not show a significant difference between groups (SMD −0.47, 95% CI −1.02–0.07; *P* = 0.09), and the heterogeneity across studies was high (τ^2^ = 0.46; χ^2^ = 29.35; *df* = 7; *P* < 0.001; *I*^2^ = 76%). Similarly, there were no differences between groups in MEP amplitude data up to 12 weeks (SMD −0.03, 95% CI −0.73–0.66; *P* = 0.92), and the heterogeneity across studies was moderate (τ^2^ = 0.24; χ^2^ = 5.38; *df* = 2; *P* = 0.007; *I*^2^ = 63%). Furthermore, the overall pooled effect showed that there was no overall effect for changes in MEP amplitude from 0 to 12 weeks (SMD −0.35, 95% CI −0.79–0.08; *P* = 0.92), and the heterogeneity across studies was high (τ^2^ = 0.39; χ^2^ = 37.39; *df* = 10; *P* < 0.001; *I*^2^ = 73%).

The MEP data between 12 weeks and 2 years (Figure 5B) showed no differences between groups (SMD −0.34, 95% CI −0.99–0.31; *P* = 0.31), and the heterogeneity across studies was high (τ^2^ = 0.43; χ^2^ = 20.37; *df* = 5; *P* = 0.0004; *I*^2^ = 80%).

### Cortical Silent Period Duration

**Figure 6:**
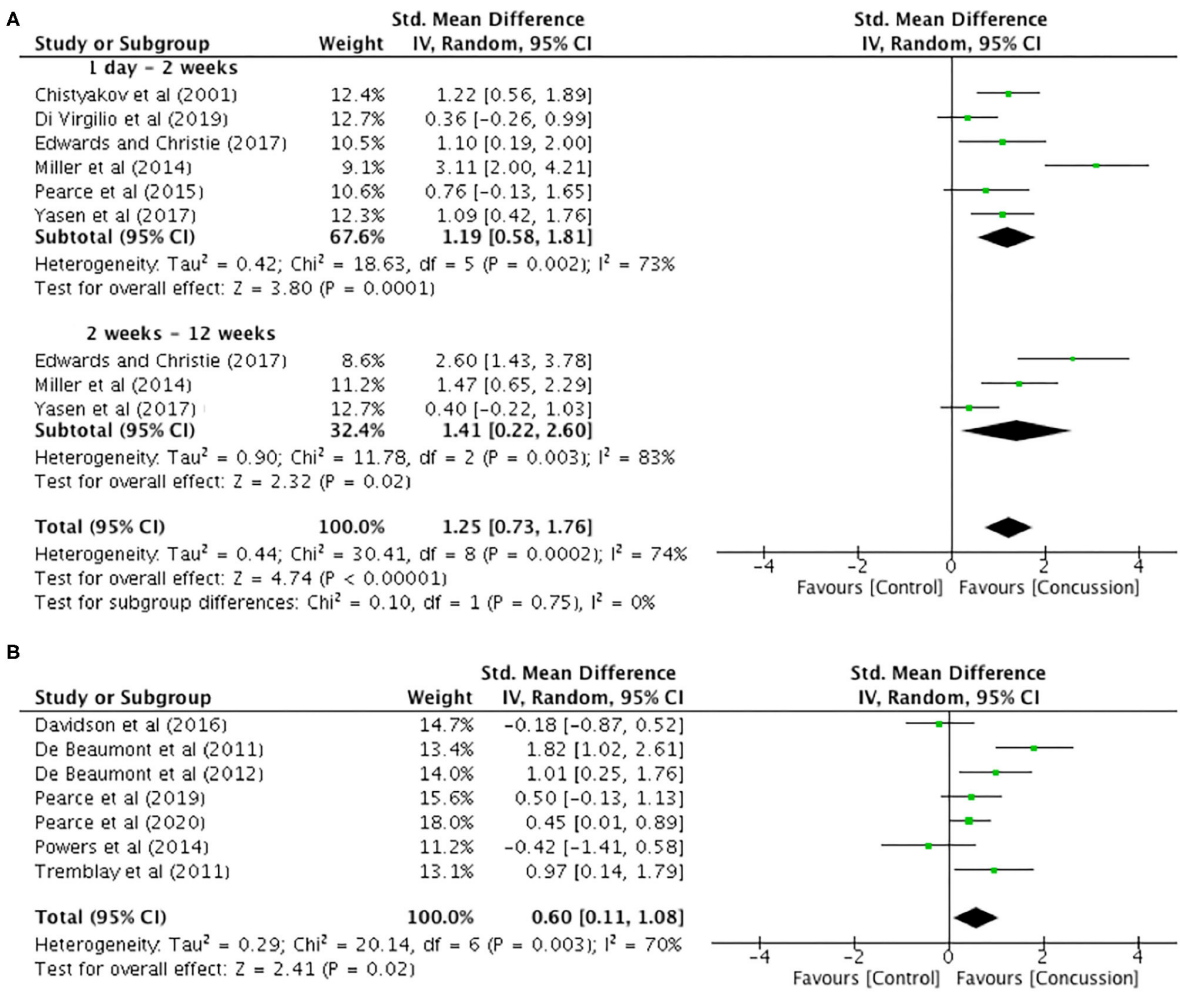
Cortical silent period duration data, 1 day−12 weeks post-concussion **(A)** and 12 weeks−2 years post-concussion **(B)**.

Between-groups data for up to 12 weeks post-concussion was extracted from six studies (Figure 6A). The post-concussion cSP data up to 2 weeks showed a significant increase in cSP duration for the concussed group (*n* = 94) compared to the control group (*n* = 104) (SMD 1.19, 95% CI 0.58–1.81; *P* < 0.001), and the heterogeneity across studies was high (τ^2^ = 0.42; χ^2^ = 18.63; *df* = 5; *P* = 0.002; *I*^2^ = 73%). Similarly, the cSP data from 2 to 12 weeks showed a significant increase in cSP duration for the concussed group (*n* = 44) compared to the control group (*n* = 49) (SMD 1.41, 95% CI 0.22–2.60; *P* = 0.02), and the heterogeneity across studies was high (τ^2^ = 0.90; χ^2^ = 11.78; *df* = 2; *P* = 0.003; *I*^2^ = 83%). The overall pooled data (0–12 weeks) also showed a significant increase in cSP duration in concussed individuals (*n* = 138) compared to controls (*n* = 153) (SMD 1.25, 95% CI 0.73–1.76; *P* = 0.02), but the heterogeneity across studies was high (τ^2^ = 0.44; χ^2^ = 30.41; *df* = 8; *P* < 0.001; *I*^2^ = 74%).

The data for 12 weeks−2 years are illustrated in Figure 6B. The data extracted from seven studies revealed that cSP duration was significantly longer in the concussed group (*n* = 128) compared to the control group (*n* = 137 SMD 0.60, 95% CI 0.11–1.08; *P* = 0.02). Moderate heterogeneity across studies was observed (τ^2^ = 0.29; χ^2^ = 20.14; *df* = 6; *P* = 0.003; *I*^2^ = 70%).

### Paired-Pulse Inhibition Measures

**Figure 7:**
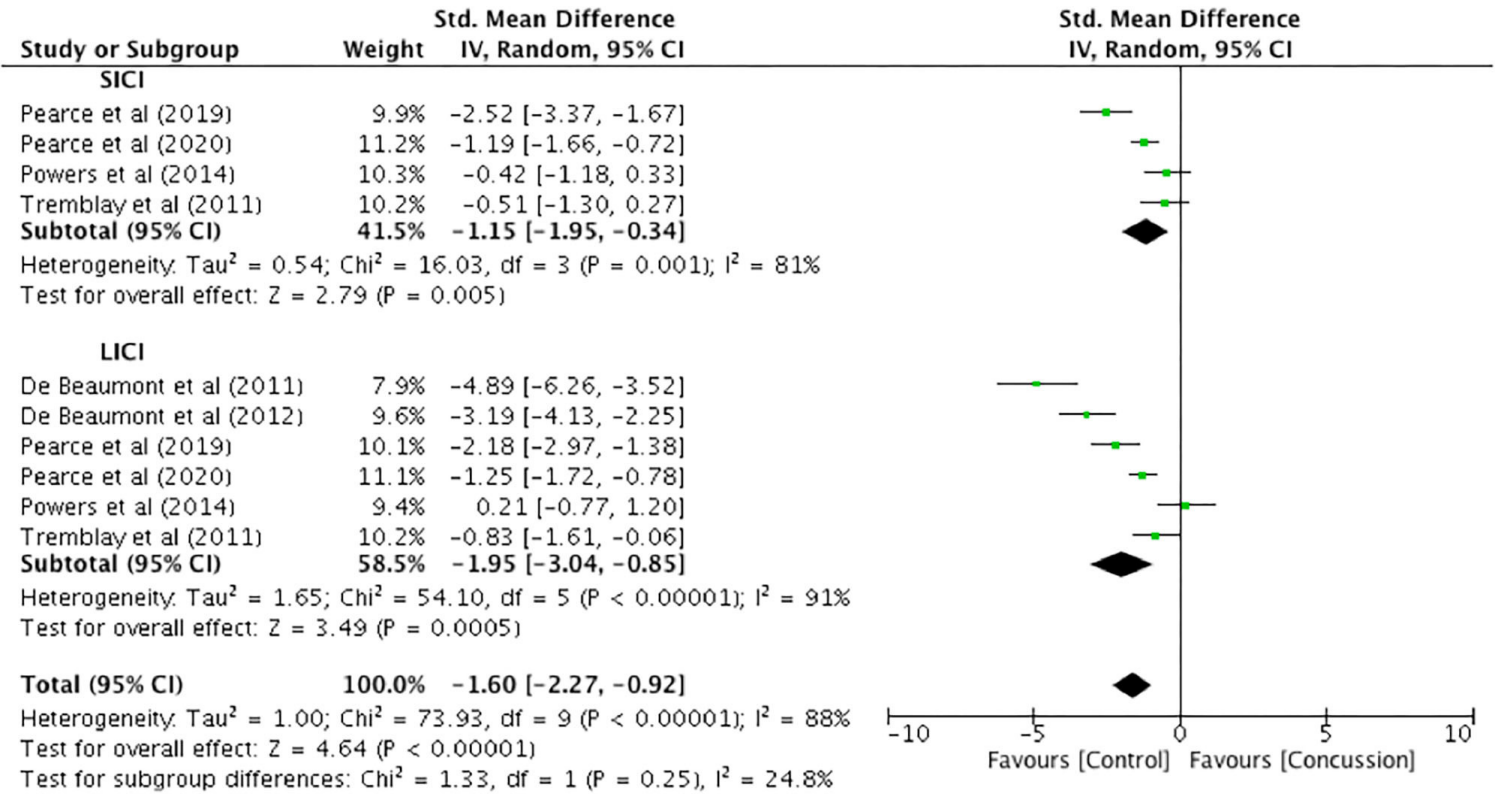
Paired-pulse (short-interval intracortical inhibition and long-interval intracortical inhibition) data, 12 weeks−2 years post-concussion.

Only one study presented paired-pulse SICI data for up to 12 weeks (Pearce et al., 2015). Therefore, meaningful analysis could not be conducted. Between-groups data from 12 weeks to 2 years post-concussion were extracted from six studies, with SICI presented in four studies and LICI presented in six studies (Figure 7). The SICI data showed a significant reduction, representing increased inhibition, in the concussed group (*n* = 115) compared to the control group (*n* = 87) (SMD −1.15, 95% CI −1.95 to −0.34; *P* = 0.005), and the heterogeneity across studies was high (τ^2^ = 0.54; χ^2^ = 16.03; *df* = 3; *P* = 0.001; *I*^2^ = 81%). Similarly, the LICI data showed a significant reduction for the concussed group (*n* = 122) compared to the control group (*n* = 124; SMD −1.95, 95% CI −3.04 to −0.85; *P* = 0.005), and the heterogeneity across studies was high (τ^2^ = 1.65; χ^2^ = 54.10; *df* = 5; *P* < 0.001; *I*^2^ = 91%). The overall pooled data (SICI and LICI) showed a significant overall decrease in paired-pulse measures in concussed individuals (*n* = 237) compared to controls (*n* = 211) (SMD −1.60, 95% CI −2.27 to −0.92; *P* < 0.001), but with high heterogeneity in the data (τ^2^ = 1.00; χ^2^ = 73.93; *df* = 9; *P* < 0.001; *I*^2^ = 88%).

## Discussion

Extending on previous systematic reviews in 2015 (Lefebvre et al., 2015; Major et al., 2015), the aim of this review was to quantify the effect of concussion injury on the corticomotor pathway via a meta-analysis. Supporting our hypothesis, the main finding from the studies included in this review showed a significant altered inhibition with increases in cSP duration and decreased SICI and LICI ratios in both acute (up to 12 weeks) and post-acute time (12 weeks−2 years) post-concussion, demonstrating reduced net corticomotor excitability. However, a concern with the data was the observation of high heterogeneity in all the measures presented, which is likely to reflect not only the methodological differences in the studies themselves but also the inter-individual response and recovery following concussion. While further research is required to build on this evidence and there are concerns with regards to the heterogeneity in responses, the data to date, from moderate- to high-quality studies with significant SMDs, suggest that TMS is an appropriate technique to assess concussion injury. Indeed the latest consensus statement includes TMS as a physiological measurement technique (McCrory et al., 2017).

Previous qualitative reviews have highlighted that the most reported changes using TMS is abnormal intracortical inhibition (Lefebvre et al., 2015; Major et al., 2015). However, this is the first meta-analysis to quantify and report significant effects, specifically the increased intracortical inhibition following concussion. Reflecting GABA_B_ receptor activity (Wilson et al., 1993), cSP duration was increased in all studies up to 12 weeks post-concussion (Figure 6A) and in all but two studies from 12 weeks to 2 years (Figure 6B). Similarly, in all but one study (Powers et al., 2014), the meta-analysis showed decreased SICI and LICI, inferring increased inhibition mediated by GABA_A_ and GABA_B_ receptor activity, respectively (Hanajima and Ugawa, 2008). While previous systematic reviews have not been able to confidently discuss intracortical inhibitory changes across cSP, SICI, and LICI, the pooled evidence from more recently published studies appear to show a strong evidence (from large pooled effect sizes) that concussion affects GABAergic neurophysiology.

It has been suggested that transient increased inhibition (24 h−10 days) following head impacts may reflect a protective reaction against minor injury (Pearce et al., 2015; Di Virgilio et al., 2016). Indeed the studies by Pearce et al. (2015) and Di Virgilio et al. (2016, 2019) have shown transient alterations in cSP duration and SICI, which return to baseline, demonstrating the dynamic nature of the corticomotor pathways. Although further studies are required, TMS may be a technique to assist in the objective determination of when an individual is fully recovered. However, future studies need to consider appropriate research methods that would allow for a suitable time course of recovery, standardised stimulus protocols, and high-quality study designs to inform clinical decisions (Kamins et al., 2017).

The results from studies investigating persistent post-concussion symptoms (beyond 3 months), showing increased intracortical inhibition, may reflect a form of maladaptive neuroplasticity in response to the injury (Bashir et al., 2010; Gosselin et al., 2012; Pearce et al., 2019, 2020a). For example, the recent studies by Pearce et al. (2019, 2020a) showed impaired reaction time performance, increased fatigue that correlated with increased cSP, and reduced SICI and LICI, suggesting that persistent symptoms have a physiological basis. This hypothesis of increased inhibition has been argued previously (Landi and Rossini, 2010; Pascual-Leone et al., 2011; Demirtas-Tatlidede et al., 2012) and posits that the increased inhibition seen in the acute phase following a brain injury is a mechanism to protect the brain in spreading further impairment. However, unlike the majority of patients who recover, in a small but notable minority, this increased inhibition does not resolve. Future studies are required to explore the underlying pathophysiology in those with persistent post-concussion symptoms to determine if factors such as previous concussion history (or history or sub-concussive head trauma from contact sports), characteristics of the injury (such as type of accident causing the concussion), and rapid management of the injury and/or rehabilitation were available. Further research using TMS along with biomarkers such as brain-derived neurotrophic factor, which plays important roles in neurone functioning, modulating neurotransmitter conduction (including GABA), and contribution to neuronal plasticity (Bathina and Das, 2015; Frazer et al., 2016), should be included in studies to fully understand the neurophysiological mechanisms contributing to persistent symptoms post-concussion. Other co-registration studies, such as TMS–EEG or TMS with neuroimaging (outside the scope of this review) will also help our understanding of the extent of injury outside of the corticospinal pathway and the mechanisms of plasticity underlying functional recovery following a concussion and a brain injury (Pascual-Leone et al., 2011; Demirtas-Tatlidede et al., 2012). Indeed TMS–EEG connectivity changes that underlie the persistent post-concussion impairments following concussion are increasingly being considered (Coyle et al., 2018), with recent evidence showing increased inhibition from TMS evoked EEG potentials (P30 and N45) following an intervention of continuous theta-burst stimulation in those with a history of concussions compared to age-matched controls (Opie et al., 2019).

Despite the excitability variables not showing significant differences, it is important to understand that, when taken in context of overall excitability, the data from this meta-analysis suggest cortical hypoexcitability. While previous studies (e.g., De Beaumont et al., 2011) and systematic reviews have attempted to implicate neuromuscular system deficits, in particular, the motor system, as contributing to an increased risk of injury (Howell et al., 2018), future studies should continue to consider the corticomotor system when attempting to answer this question.

### Limitations of the Current Research and Suggestions for Future Studies

This systematic review and meta-analysis focused on non-intervention acute and post-acute studies (up to 2 years) using specifically TMS–EMG. As a result, techniques such as PAS, repetitive TMS (including theta-burst protocols), and transcranial direct current stimulation (tDCS) did not meet our inclusion criteria. However, this is not to say that differences between concussed individuals and age-matched controls have not been reported. For example, PAS has been utilised in comparing previously concussed athletes (mean time post-concussion, 13.7 ± 6.2 months) to age-matched athletes with no history of concussion (De Beaumont et al., 2012b). These authors reported that, compared to controls, previously concussed athletes demonstrated an increased inhibition following the PAS intervention. Repetitive stimulation studies (theta-burst, tDCS) have been limited and have mixed results. In young adults who reported a history of concussion in adolescence, Meehan et al. (2017) showed, following an intermittent theta-burst intervention protocol, that MEP amplitude and intracortical facilitation were lower and SICI changes were more variable in the concussion history group. Research investigating the effects of tDCS in those with a history of concussion (mean time post-injury, 21.2 ± 13.5 months) revealed no change in resting motor threshold or cSP duration post-tDCS intervention (Wilke et al., 2017). Interestingly, while transcallosal inhibition differences have been reported in those with a chronic history of concussions, i.e., >2 years post-concussion (Davidson and Tremblay, 2016), there has been no studies using interhemispheric inhibition (IHI) technique. Our search did uncover one study reporting no difference in IHI between concussed and control groups (Locke, 2019); however, this was a Master's thesis and therefore did not meet our inclusion criteria.

Another limitation in this review, which reflects the research into acute, post-acute, and long-term outcomes more generally, is that we could not analyze TMS data with regards to gender, asymptomatic vs. symptomatic, and the quantified number of concussions reported. Emerging evidence is suggesting that, following a concussion, females have greater severity of symptoms and may take longer to recover (Koerte et al., 2020); however, TMS studies have not specifically investigated this question by providing gender-specific TMS data. Similarly, an investigation of the number of concussions experienced has been limited with groups divided between “history of concussion” and “no history of concussion.” One study (De Beaumont et al., 2007), however, did compare multiple concussions (mean 2.7 ± 1.3) to those with only one reported concussion and age-matched controls, showing no differences in motor threshold, MEP amplitude, or cSP duration between concussed groups, with differences only being observed between both concussed groups to controls. Finally, TMS studies have generally compared “concussed” (including those with a history of concussion) to “non-concussed” controls. To date, only two TMS studies from the one group (Pearce et al., 2019, 2020a) has compared three cohorts (symptomatic persistent post-concussion symptoms, asymptomatic post-concussion, and age-matched controls), reporting that those with ongoing symptoms had increased cSP duration and decreased SICI and LICI compared to asymptomatic and control participants. Collectively, these limitations should inform future study designs.

## Conclusion

Developed 35 years ago, TMS has consistently been demonstrated as a reliable and sophisticated technique in neurophysiology research that can detect subtle changes in the neurological system in healthy individuals and those with a variety of neurological impairments (Kobayashi and Pascual-Leone, 2003; Lanza et al., 2017; Pennisi et al., 2017). While TMS studies into concussion are emerging, the data from this systematic review and meta-analysis illustrate that TMS is not only a technique that can identify physiological markers following a concussion and provide return-to-full-activity decision but also a tool that has potential detection of underlying sub-clinical mechanisms in those with persistent symptoms. However, further studies are required to establish the clinical efficacy for a systematic application of TMS as a diagnostic tool for concussion and mild brain injury. While studies in this review were rated as moderate to high in TMS quality, one suggestion toward improving wider clinical confidence in TMS is to have a consensus on methodological consistency and improved designs to reduce the risk of bias. Nonetheless, the potential of TMS to reliably quantify cortical activity offers important opportunities to provide a low-cost, objective biomarker to value-add to existing clinical assessments of concussion.

## Data Availability Statement

The raw data supporting the conclusions of this article will be made available by the authors, without undue reservation.

## Author Contributions

ES and AP contributed to the study concept, design, and data collection. DK and AF contributed to the study quality assessment. All authors contributed to the drafting of this article.

## Conflict of Interest

AP currently received partial research salary funding from Sports Health Check charity (Australia). AP has previously received partial research funding from the Australian Football League, Impact Technologies Inc. (Australia), and Samsung Corporation and has provided expert testimony to courts on concussion injury. The remaining authors declare that the research was conducted in the absence of any commercial or financial relationships that could be construed as a potential conflict of interest.
